# Enhanced corticospinal excitability in the tibialis anterior during static stretching of the soleus in young healthy individuals

**DOI:** 10.1371/journal.pone.0284289

**Published:** 2023-04-11

**Authors:** Francesco Budini, Monica Christova

**Affiliations:** 1 Institute of Human Movement Science, Sport and Health, Graz University, Graz, Austria; 2 Otto Loewi Research Center, Physiology Section, Medical University of Graz, Graz, Austria; 3 Institute of Physiotherapy, Institute of Applied Sciences FH-Joanneum, Graz, Austria; Università Campus Bio-Medico di Roma, ITALY

## Abstract

Corticospinal excitability is known to be affected by afferent inflow arising from the proprioceptors during active or passive muscle movements. Also during static stretching (SS) afferent activity is enhanced, but its effect on corticospinal excitability received limited attention and has only been investigated as a single average value spread over the entire stretching period. Using transcranial magnetic stimulation (TMS) the present study was conducted to explore the time course of corticospinal excitability during 30 seconds SS. Motor evoked potentials (MEPs) after TMS were recorded from soleus (SOL) and tibialis anterior (TA) muscles in 14 participants during: a passive dynamic ankle dorsiflexion (DF), at six different time points during maximal individual SS (3, 6, 9, 18, 21 and 25 seconds into stretching), during a passive dynamic ankle plantar flexion (PF) and following SS. To explore the time course of corticospinal excitability during the static lengthened phase of a muscle stretch, the stretching protocol was repeated several times so that it was possible to collect a sufficient number of stimulations at each specific time point into SS, as well as during DF and PF. During passive DF, MEPs amplitude was greater than baseline in both TA and SOL (p = .001 and p = .005 respectively). During SS, MEPs amplitude was greater than baseline in TA (p = .006), but not in SOL. No differences between the investigated time points were found and no trend was detected throughout the stretching time. No effect in either muscle was observed during passive PF and after SS. These results could suggest that an increased activity of secondary afferents from SOL muscle spindles exert a corticomotor facilitation on TA. The muscle-nonspecific response observed during passive DF could instead be attributed to an increased activation within the sensorimotor cortical areas as a result of the awareness of the foot passive displacements.

## Introduction

In the last few years, an increasing number of investigations tested the effects of stretching on spinal and corticospinal excitability, to explore whether the commonly observed stretching-induced decrease in physical performance can be attributed to neuromuscular factors [1–6]. Indeed, during both the dynamic and static phase of a stretch, muscle spindles, joints, and skin receptors are stimulated [7,8] and their afferents project to the cortex [9–12], potentially affecting force output. When testing the effect of stretching on spinal and corticospinal excitability however, three different phases should be considered separately and together, as each phase can influence the next phase, as well as the final value measured after stretching. These phases are: 1) the elongation phase needed to bring the muscle to the stretched position, 2) the static phase when the muscle is kept in the elongated position, and 3) the shortening phase when the joint is brought back to its original position and the muscle to its original length.

The effects of stretching phases at central level can be evaluated noninvasively using transcranial magnetic stimulation (TMS). The magnetic stimulation induces neural axon depolarization in the primary motor cortex (M1), which can be recorded as motor evoked potential (MEP) in corresponding peripheral muscles using surface electromyography [13]. The TMS-induced MEP and its related parameters reflect cortical, subcortical and spinal excitabilities [14].

Using TMS, corticospinal excitability has been studied during lengthening and shortening movements for over thirty years. Results for the arm and forearm muscles are generally consistent and demonstrate, with few exceptions [8], a depression of corticospinal excitability during lengthening and an increase during both shortening passive movements [15–17] and active contractions [15,18,19]. In the lower limb musculature, the results are more susceptible to the muscle tested, the contraction type and intensity, and the age of the participants [20–24], but in general all these studies seem to indirectly confirm that variations in peripheral afferent input could affect pathway excitability [25,26].

During static stretching (SS), the amplitude of MEPs was reported to be reduced compared to baseline when the ankle joint was kept at 20° dorsiflexion (DF), but not when it was kept at 10° DF [27], which would suggest that: 1) the lengthening displacement, with related increased Ia afferent activity, does not have an effect lasting enough to influence corticospinal excitability during the following static stretching, and 2) that possibly also muscle spindle secondary afferents influence corticospinal excitability.

Following stretching, with the muscle at rest, a very short (circa 2 seconds) facilitation in corticospinal excitability was observed [28], but when looking at an average value spread over longer time windows, no changes in MEP amplitude were reported [1,29,30], and even a reduction of MEP variability was observed [31]. During muscle contractions, results are conflicting, with maximal contractions and contractions at 20% of maximum yielding no changes [1,32], whilst an increase in corticospinal excitability was observed during contractions at 30% of maximal [33].

To better understand and to complete this still not fully clear picture of the corticospinal changes in response to stretching several key components are missing, one of these being the limited information on the time course and instant values of the observed responses. Indeed, examining the neuromuscular effects of stretching by looking at an average value representative of a during- or an after-stretching phase, restricts the possibility to deeply understand the ongoing neurophysiological mechanisms [34]. This limitation is caused by two methodological requirements related to the assessment of spinal and corticospinal excitability, namely: having to collect and average several stimulations and having to allow several seconds between one stimulation and the next [35]. As a result, the majority of the studies describe phenomena that do not represent instant time points, but average values spread over a time window of several seconds.

Acknowledging the limited ecological relevance of this issue when referring to sport and rehabilitation practices, from a strictly neurophysiological point of view, the possibility to detect a trend, or a specific time course of a response, may contribute to comprehend its origin and its implications. Moreover, as already mentioned, it has to be considered that for measuring a parameter at the static end position of a stretch, and after it, a limb needs to be displaced to reach this position and then return from the stretched to a neutral position. Such lengthening and shortening displacements could influence the measurements collected during the stretch (following lengthening) and after the stretch (following shortening) [15–18,36,37]. One way to detect whether this has occurred, is by repeating the stretching protocol several times and repeat several times the stimulation at specific time points into and after the stretch. This allows to distinguish between a potential long-lasting effect of the lengthening and shortening displacement, as well as to monitor the time course of the response throughout the stretching period when the muscle is maintained in an elongated position. Such methodology, however, is not commonly adopted in studies investigating stretching. Only recently we implemented this approach to study in detail the time course of spinal excitability at the static end position of a stretch and after stretching [28,38] as well as the time course of corticospinal excitability following stretching [28]. However, to our knowledge, the behaviour of corticospinal excitability throughout the stretching time has never been investigated. For example, it is still unknown whether the inhibition of MEPs amplitude observed by Guissard and colleagues at 20° SS [27] remained unaltered throughout the stretching time or showed a tendency to recover, as for example observed for spinal excitability [38]. Moreover, it is unknown whether the inhibition persists during the passive shortening phase back to the tested ankle neutral position and whether the passive plantarflexion (PF) contributes to the recovery or even promotes the facilitation detected within the first 2 seconds immediately after stretching [28].

The current TMS study was conducted to investigate the time course of corticospinal excitability of plantar flexors at the static end position of a stretch. The passive lengthening and shortening phases were also monitored to obtain a clearer and more comprehensive picture.

## Methods

### Participants

Fourteen recreational active individuals: 5 males (age 23.6 ± 1.5 years, body mass 75.2 ± 7.3 kg, stature 180 ± 7.9 cm) and 9 females (age 24.3 ± 2.0 years, body mass 58.0 ± 3.0 kg, stature 166 ± 4.8 cm) were recruited for this study. Volunteers abstained from any physical activity within 24 h before the testing. The study was approved by the Ethics Committee at the University of Graz (GZ. 39/77/63 ex 2013/14), all experiments were performed in accordance with relevant guidelines and regulations and written informed consent was obtained from all volunteers before the onset of the experimental procedures.

### Study design

Participants attended the laboratory on two separate occasions: the first, lasting about 90 minutes, for familiarising with testing procedures and equipment and the second for the actual testing session. The testing sessions lasted about two and half hours and were run in the morning (13 out of 14 participants started before 1pm and only one started in the afternoon at 4pm).

The experiment consisted in the measurement of MEPs after TMS during: during passive dynamic ankle DF, at different time points during SS, during a passive dynamic ankle PF and following stretching.

Ten TMS stimulations were performed at each of the following time points into stretching: 3, 6, 9, 18, 21 and 25 seconds in random order. Moreover 16 MEPs were elicited during dynamic DF and 14 during dynamic PF. Further stimulations were performed between 10 and 20 seconds at the end of the stretching procedure.

### Experimental procedures

The experimental procedures have already been described in our previous works [28,38]. Subjects were sitting on an isokinetic dynamometer (CON-TREX MJ, CMV AG, Duebendorf, Switzerland) with the standard setup for ankle joint rotation individually adjusted. Participants had their right knee fully extended and the foot resting on the dynamometer footplate, the ankle joint aligned with the dynamometer rotation shaft and the ankle angle set at 100° (10° plantar flexion deviating from a neutral position at 90°). Volunteers sat with the trunk at 110° and the head supported by a cushion (dentafix^®^, pro medico HandelsGmbH, Graz, Austria) that once positioned could be deflated allowing the formation of a stable form molded on the volunteers´ head and neck shapes. By using a remote control, the volunteers were instructed to adjust the dorsiflexion isokinetic rotation operated by the dynamometer around the foot plate until the point of perceived maximal DF (therefore the maximal dorsiflexion was not fixed at a specific value, but was different for each volunteer). Participants were asked to keep their knees extended and to relax during the procedures.

Once the maximal individual dorsiflexion was defined, participants were prepared for surface electromyographic recording (EMG) from tibialis anterior muscle (TA) of both right and left leg as well as from the right soleus muscles (SOL). The volunteers then returned to sit on the dynamometer chair in the position described above and were instructed to relax meanwhile coil position and stimulation intensity for TMS was determined. Subsequently a trigger-driven sequence of stimulations started and continued for 150 seconds until 30 MEPs were collected for baseline reference values.

**Fig 1:**
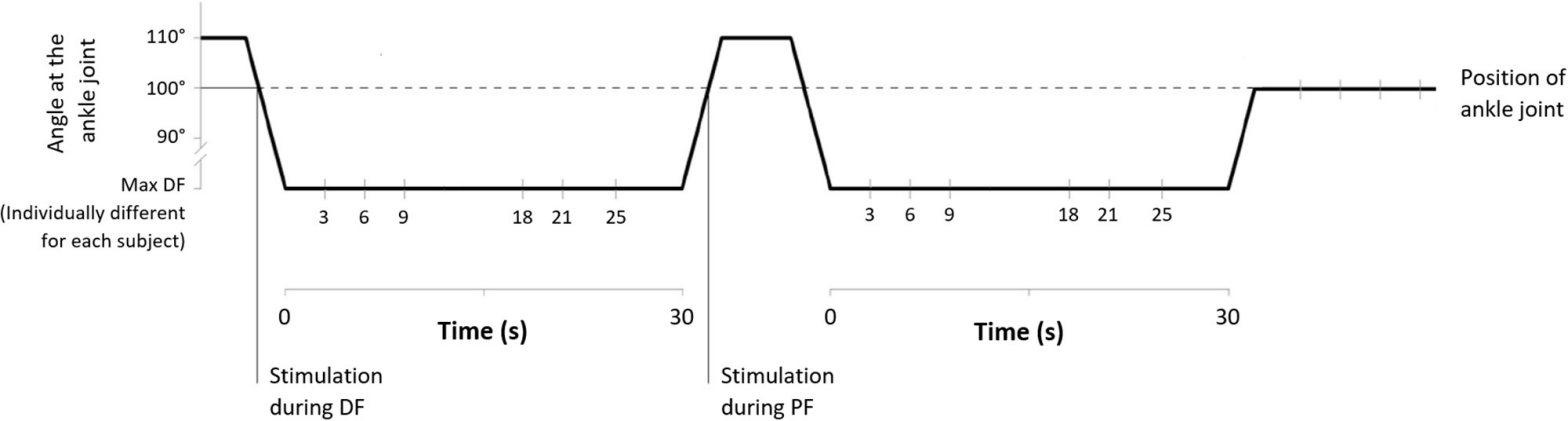
Stretching protocol and stimulation points.

In order to perform all measurements at 100° during dynamic PF, following baseline recordings, the foot was passively rotated to a starting position of 110° plantar flexion. In this way the stimulations during dorsiflexion movements (20°/s) could be delivered when the ankle reached 100° PF (Fig 1).

The sequence of stimulations during the intervention is represented in Fig 1. A first stimulation was delivered during dynamic DF, then, once individual maximal dorsiflexion was reached, the position was kept for 30 s and during this period a second stimulation was delivered at one of the investigated time points (3, 6, 9, 18, 21 and 25 s into stretching). After this first 30 s SS bout, the foot was passively rotated (20°/s) back to 110° PF and a third stimulation was delivered during the PF movement as the ankle was at 100° PF. This procedure was repeated without pauses a second time with only one more stimulation delivered at maximal dorsiflexion and, after the second 30 s stretch, further stimulations were performed with the ankle joint back at 100°.

To collect a sufficient number of stimulations at each given time point, this 2 x 30 s stretching block was repeated 30 times; 50 second rest with the volunteer sitting were allowed between every block and two minutes rest with the volunteer standing up every 12 stretching applications.

### Surface electromyography

For preparation of EMG recording, skin was shaved, abraded, cleaned with alcohol, and left to dry. Surface EMG was collected from the SOL and TA of the right leg. Additionally, the EMG signal was recorded from the tibialis anterior of the left leg to ensure the correct directional positioning of the coil. Electrodes (Blue Sensor N, Ambu A/S, Ballerup, Denmark) were placed in monopolar configuration for recording from the SOL muscle (as suggested by Hadoush et al. [39]); the electrodes for TA were placed in bipolar configuration with an interelectrode distance of 20 mm. One ground electrode per leg was placed over the tibial bone medial surface.

### Transcranial magnetic stimulation

All stimulations at baseline, during dynamic DF and PF and after stretching were performed with the ankle joint at 100° PF, stimulations during stretching were performed at individual maximal DF position (Fig 1).

Motor evoked potentials in response to single pulse TMS were induced by a Magstim 200, (Magstim Company Ltd., UK) using a double cone coil (110mm coil diameter). The coil was placed over the M1 of the leg area, 1–2 cm posterior from the vertex and slightly shifted to the left side in order to obtain the largest response from the contralateral right SOL. Resting motor threshold was determined as the minimum stimulator intensity able to evoke MEPs of at least 50μV amplitude in more than five out of ten consecutive trials [13]. To ensure a constant coil positioning throughout the experiments, subjects were wearing an electroencephalography cap on which the optimal coil position was marked with a soft pen. MEPs were elicited at 5-s intervals with stimulation intensity equal to 120% of the resting motor threshold.

### Data monitoring

EMG was monitored online to control that the subjects were not actively contracting their plantar flexors against the stretch. However, a more careful control of this parameter was then repeated off line during data analysis (see *Data analysis*).

During the recordings, the trigger signal responsible for the activation of the TMS was simultaneously sent to the A/D converter. This served off line for data analysis and online for signal monitoring, as the A/D software (DeweSoft) was programmed similarly to an oscilloscope to freeze the screen within a time window of 80ms before and 80ms after the trigger event to give the experimenter the possibility to control every single MEP. Additionally, 2 cursors were placed on the screen approximately in correspondence with the MEPs average value; this allowed to detect any extra-ordinary MEP, and, in case, lead to the decision of collecting extra data for that specific series. The variability of the measures was additionally checked offline (see *Data analysis*), therefore, the extra recording, when done, only increased the chance to retain more MEPs within the acceptable variability range for the subsequent analysis.

### Data analysis

Electromyography, foot displacement and trigger signals were synchronized (Dewetron 7.0 recording system), digitized with a sampling frequency of 10 KHz, stored on a PC and analysed using custom algorithms developed in Matlab (R2014b).

Baseline MEPs with peak to peak amplitude exceeding by ±2 standard deviations the average baseline value were discarded [40]. Similarly, consistency was checked within the 60 MEPs (10 stimulations at each of the 6 time points) collected during stretching and the MEPs collected during DF and PF (16 and 14 MEPs respectively). Because voluntary activation increases MEP amplitude [41], additional discards were applied in all those cases where MEPs were elicited in a pre-activation state of the target muscle (defined as EMG activity larger than 50 μV in the 50 ms preceding the TMS pulse) [42]. All the remaining waves were retained and when a minimum number of 5 MEPs per data set was still available, the average value was used for statistical analysis.

### Statistical analysis

Measurements were checked for normal distribution by the Shapiro-Wilk test. Since data were not normally distributed, Friedman test followed by Wilcoxon signed-rank tests with Bonferroni Holm’s adjustment was used to compare values at baseline, during dynamic DF, during SS (all values merged), during dynamic PF and post stretching. The effect sizes (ES) were calculated for every comparison done with the Wilcoxon signed-rank tests that was still significant following Bonferroni Holm’s adjustment. These were calculated dividing the *z* value by the root square of the product: conditions *x* observations [43].

The time course of MEPs amplitude throughout the stretching was tested by a Friedman test to compare values at different time points into stretching and further assessed with Pearson ’s correlation for linear regression analysis.

All statistical analysis was completed using PASW Statistic 18.0.0.

## Results

Transcranial magnetic stimulation was well tolerated by all participants, stimulus intensity at resting motor threshold was 45±6% of the maximum stimulator output and no side-effects were reported. One subject was excluded for methodological problems. During SS, five volunteers showed pre-activation of the SOL muscle before several MEPs, consequently not a sufficient number of MEPs could be retained for subsequent analysis of specific time points into stretching, the analysis was therefore performed on the remaining 8 participants. However, for the comparison baseline vs SS, when all time points into stretching were averaged, we could retain data from 10 subjects. Data from 11 subjects could be retained for comparisons vs PF and from 13 subjects for comparisons vs DF and vs Post. For TA no participants were excluded.

**Fig 2:**
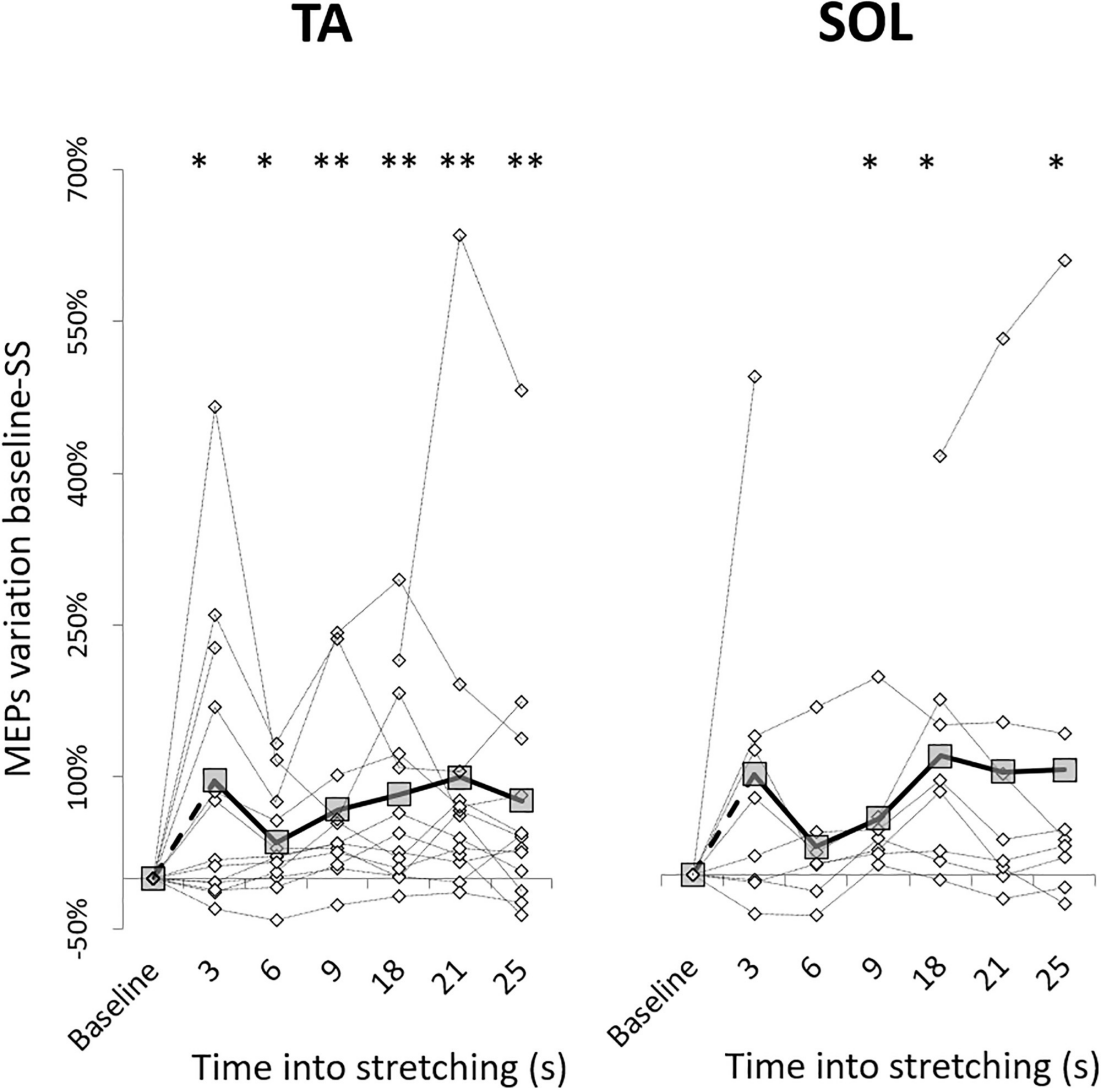
Time course of MEPs facilitation during stretching. Group average (thick line with squares) and individual (thin dotted line with diamonds) are expressed as percentage of variation from baseline values. Some values overlap. Comparison to Baseline: * p < .05; ** p < .01.

During static stretching the amplitude of the evoked MEPs was greater than at baseline for both SOL (χ^2^_3_ = 16.393, p = .012) and TA (χ^2^_3_ = 19.036, p = .004) at every tested time point for the TA, but only at 9, 18 and 25 seconds into stretching for the SOL (at 3 and 6 seconds p = .069, at 21 seconds p = .086) (see Table 1 for the effect sizes). No differences were observed between different data points into stretching (SOL: χ^2^_3_ = 11.071, p = .05 (no pair differences after Bonferroni Holm’s correction) and TA: χ^2^_3_ = 7.714, p = .17) and also no correlations between time into stretching and MEPs amplitude were detected for both SOL and TA (p = .54 and p = .78 respectively) (Fig 2). Consequently, all the MEPs elicited during stretching were averaged together.

**Table 1:** p-values, z-values, and effect sizes of the statistically significant comparisons.

Comparison for TA	P-value	Z-value	Effect Size
DF Vs Baseline	0.001	-3.180	-0.62
SS Vs Baseline	0.006	-2.760	-0.54
DF Vs PF	0.002	-3.110	-0.61
DF Vs Post	0.001	-3.180	-0.62
3s Vs Baseline	0.016	-2.411	-0.47
6s Vs Baseline	0.028	-2.197	-0.43
9s Vs Baseline	0.005	-2.824	-0.55
18s Vs Baseline	0.004	-2.900	-0.57
21s Vs Baseline	0.003	-2.970	-0.58
25s Vs Baseline	0.006	-2.760	-0.54
Comparison for SOL	P-value	Z-value	Effect Size
DF Vs Baseline	0.005	-2.830	-0.55
9s Vs Baseline	0.025	-2.240	-0.56
18s Vs Baseline	0.025	-2.240	-0.56
25s Vs Baseline	0.025	-2.240	-0.56

TA = Tibialis Anterior; SOL = Soleus; DV = dorsiflexion; SS = Static Stretching; PF = Plantar flexion.

**Fig 3:**
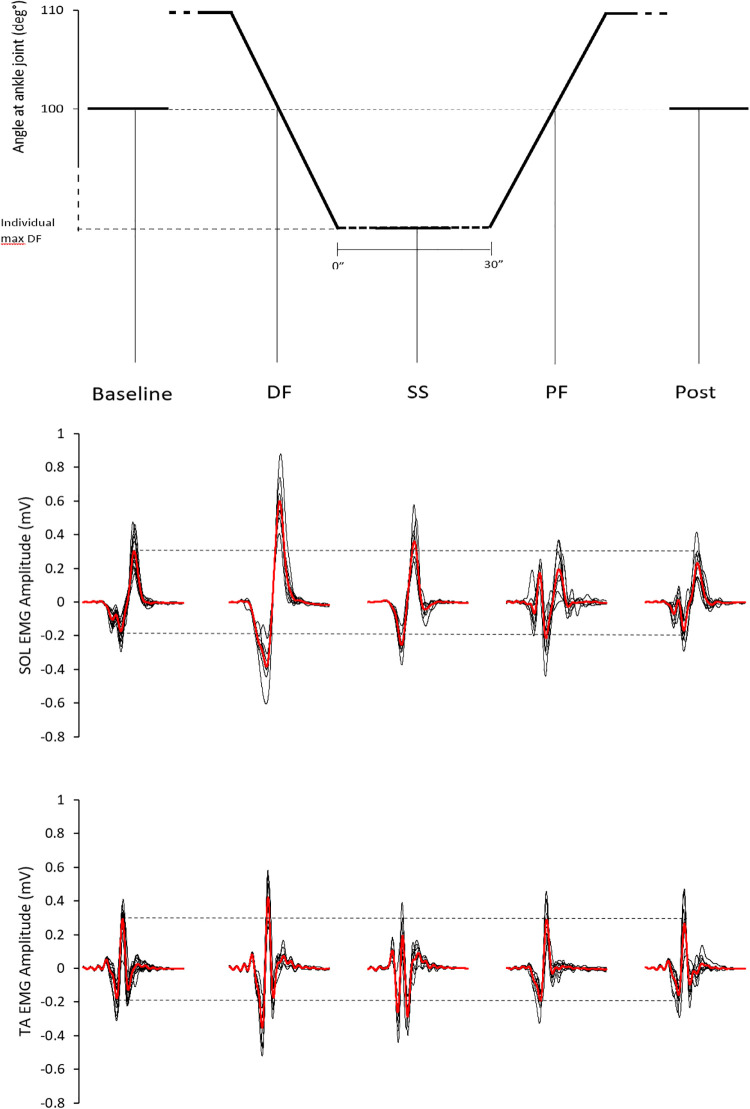
Single subject example for MEPs. On the upper part of the graph is outlined the angle at the ankle joint at which the stimulations were delivered. In the middle and lower part of the graph are presented the SOL and TA EMG tracks recorded at: Baseline, during DF, during SS, during PF and after stretching. For baseline circa 15 waves are superimposed (black waves), for all the other stimulation points circa 10 waves are superimposed. The thicker red waves represent the average values and the dashed horizontal lines limit the positive and negative peak of the average baseline value. For graphical purposes raw data was down sampled and low passed filtered.

**Fig 4:**
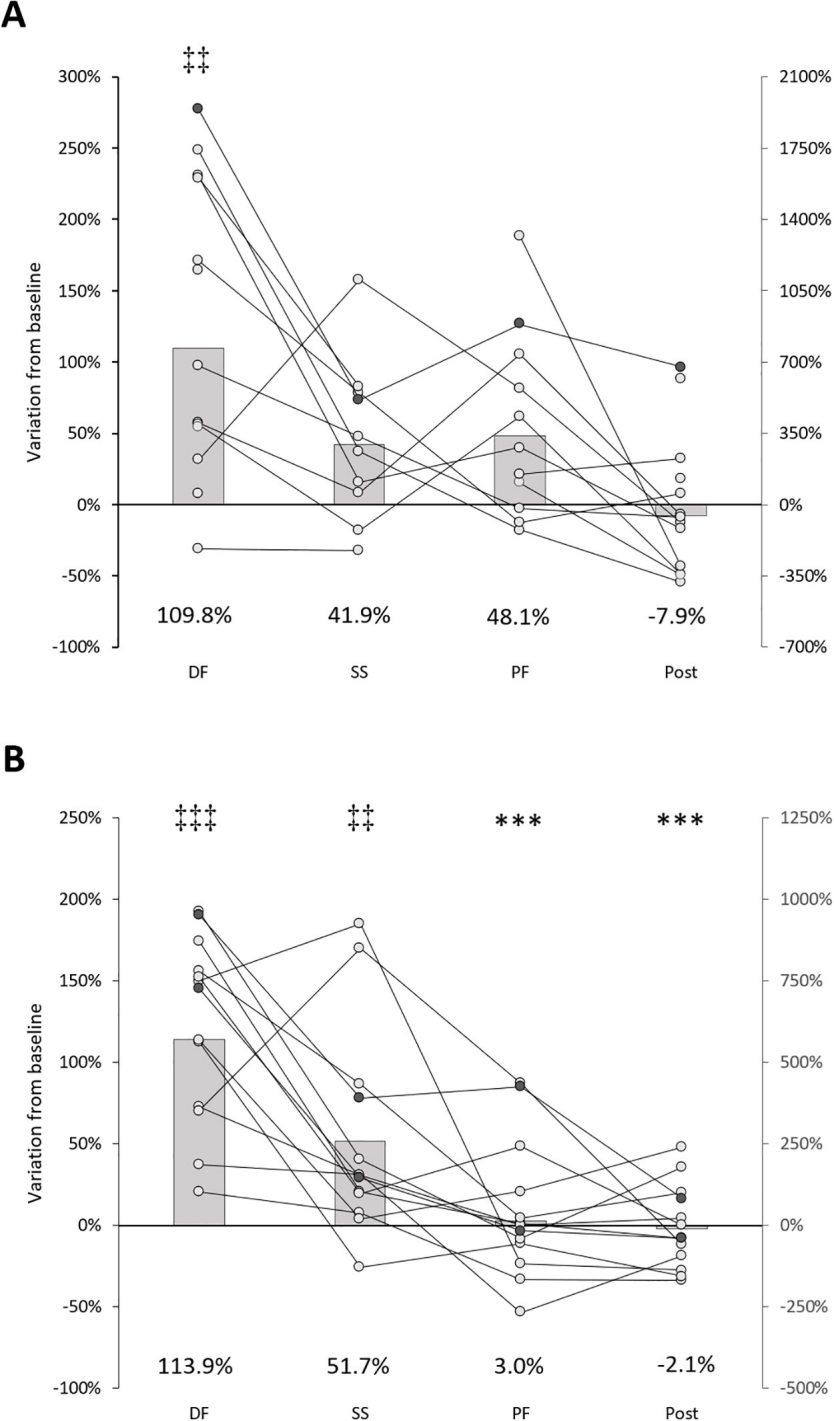
A: SOL; B: TA. On the primary axis, bars: Group average MEPs amplitude expressed as percentage of baseline values (with data labels); connected dots: Invidividual values. On the secondary axis are plotted the individual subjects represented with thicker lines and filled circles (for graphical reasons these values were not included for computing the group average bars). Comparison to DF: *** p < .005; Comparison to baseline: ‡‡ p < .01; ‡‡‡ p < .001.

Fig 3 shows MEPs data from a representative participant at baseline, during dynamic DF, during SS (random time points), during dynamic PF and post stretching. As visible from the figure, for both SOL and TA, the amplitude of the MEPs evoked during the DF passive movement is clearly bigger compared to the amplitude of those MEPs recorded at the other stimulation points (Fig 3). Group average values (presented as percentage of variation from baseline values in Fig 4) reflect the data of the representative participant. There was a statistically significant difference between the amplitude of the MEPs evoked in the different conditions for both SOL (χ^2^_3_ = 22.489, p = 0.00016) and TA (χ^2^_3_ = 30.533, p = .000004) with higher MEPs amplitude during dynamic DF compared to baseline for both muscles (for SOL: Z = -2.830, p = .005, ES = -.55; for TA: Z = -3.180, p = .001, ES = -.62). Additional pair comparisons remained statistically significant after Bonferroni Holm’s correction for TA (DF vs post: Z = -3.180, p = .001, ES = -.62, DF vs PF: Z = -3.110, p = .002, ES = -.61, and SS vs baseline: Z = -2.760, p = .006, ES = -.54,), but not for SOL.

## Discussion

The aim of the present study was to examine the time course of corticospinal excitability when the plantar flexors were statically kept in an elongated position (i.e., during SS) as well as to monitor potential variation during dynamic DF and PF. For both SOL and TA, the amplitude of the MEPs evoked during DF was significantly bigger compared to baseline. Throughout the entire 30 sec SS the corticomotor excitability was significantly increased for TA and did not show a time-dependent trend.

### Response modulation during SS

Cortico-spinal excitability is affected by muscle length and limb posture [44–49], and such alterations are commonly attributed to afferent projections to cortical and/or spinal paths [17,50]. Potential sites of modulation of the MEP in response to TMS include intrinsic cortical inputs to the pyramidal tract neurons in M1, the activity of the α-motoneurons and interneuron network at spinal level and the afferent signals arising from activated sensory receptors. At least in the upper limb, MEPs amplitude tends to increase when the muscle is at a shorter length. For example, greater MEPs in the biceps brachii are observed when this is in a shorter position with the forearm supinated, compared to when the biceps brachii is in a lengthened position, with the forearm pronated [45–47], and this effect seems related to the amount of stretch [49]. Although results on the lower limb don´t always corroborate this observation [44], our data confirm that TA cortico-spinal excitability increases when the muscle is shorter. Additionally, the present study tried to extend the current knowledge by investigating whether this expected facilitation in the shortened muscle would have remained constant throughout the entire stretching period or not. MEPs were therefore collected at six time points: 3, 6, 9, 18, 21 and 25 seconds into stretching. Since we observed a clear facilitation during the DF movement required to bring the foot to maximal individual static stretched position, we could have expected to capture a transient effect within the first measurement at 3 seconds. Looking at Fig 2 this seems to be the case only in 6 of the 13 subjects. Although, the group average seems to show a pattern consisting in an initial sharp facilitation at 3 seconds into stretching, followed by a reduction of this from 3 to 6 seconds, and finishing with a more or less linear increase, this pattern is mostly affected by 3 subjects who had remarkable changes from baseline to 3 seconds into stretching. The absence of a time-dependent trend in the increased excitability is confirmed by the absence of statistical differences between time points into stretching.

### Response modulation during dynamic DF

The observed corticomotor facilitation during DF was registered for both the elongated (SOL) and the shortened (TA) muscles. While the increased corticomotor excitability of TA during DF is in line with studies showing an increased MEP amplitude during passive muscle shortening [15–19,23], the simultaneous facilitation in the elongated muscle SOL is not supported by previous findings, which commonly report reduced MEP amplitude during lengthening of the wrist and elbow flexors and extensors [15–19,51]. However, differently to the upper limb, responses in the lower limb are sometimes contradictory being susceptible to more variables [20–24]. For example, Škarabot and colleagues reported a facilitation in the shortened TA in young [23] but not in old participants [24] and no effect on the lengthened SOL [23,24], whilst Hultborn and colleagues [8] did not report any facilitation in neither TA nor SOL, and in the present study we witnessed a facilitation in both TA and SOL.

Considering that TMS activates predominantly monosynaptic pathways to the α-motoneurons and these connections are not exposed to presynaptic inhibition [17], it seems that the observed excitability alterations during DF can be attributed either to cortical inputs to the corticospinal pathways or to postsynaptic inputs directly modulating the α-motoneurons. However, a recent study showed that during passive ankle movements, the excitability at the lumbar spinal segmental level was not modulated in neither TA nor SOL [23], suggesting supraspinal rather than postsynaptic contributions to the observed MEP facilitation.

Supraspinal structures can indeed be affected by proprioceptive afferents [17,50]. During passive lengthening movements, Ia fibres from muscle spindles respond to changes in muscle length by increasing their firing rate [52,53]. The respective inflows projecting primarily to the area 3a in the primary somatosensory cortex (S1) [54], can activate indirectly the cortical inputs to the pyramidal tract neurons in M1. Such co-activation within the S1 area and the motor areas (M1 and SMA) in response to passive proprioceptive stimulation (passive fingers’ flexion and extension) was demonstrated in a fMRI study [55]. It is known that the finely scaled topographic maps of S1 and M1 enable both areas to have highly specialized responses to changes in the periphery [56,57]. As shown in a sensorimotor slice [58], the anatomical and functional sensorimotor connections are reciprocal and their localization in cortical layers V and VI allow descending outputs also to the spinal cord [59]. In our study we can expect that the increase in Ia afferents activity from SOL during DF reached the somatosensory area (3a), which transmits these inputs further to the M1 output neurons, increasing the excitability of the descending corticomotor pathway.

Such increased excitability originating from the cortex with related facilitation on the descending drive, could be a compensation mechanism for the spinal inhibition (typically observed as decrease in H-reflex during lengthening movements for review see [34]), as shown in a study with active muscle lengthening [19]. However, if an increased Ia activity provoked an increase in corticospinal excitability or a reduction of cerebello-cortical inhibition on projections on the homonymous muscle, we should have observed not only greater MEPs in the SOL during DF, but also in the TA during PF movement. Alternatively, in case of projections on the antagonist muscles, we should have observed greater MEPs in the TA during DF together with greater MEPs in the SOL during PF movement, but neither of these two scenarios was the case (Fig 4).

A possible explanation of this not muscle-specific response could be attributed to the awareness of the subjects about the passive displacements of the foot, that, for methodological requirements, was repeated 60 times. Indeed, in healthy individuals, passive movements are shown to activate not only the primary somatosensory cortex but also the primary motor cortex, supplementary motor area, and posterior parietal cortex as well as the secondary somatosensory cortex (S2) (for review see [60]). Moreover, passive movements can selectively increase cortical excitability in relation to the duration and velocity of the movements, the presence of rest, and whether attention is directed to the movement. Attention to the stimulated side during an intervention (peripheral afferent stimulation, passive movements) decreases the activity of the inhibitory cortical circuits, and thus increases corticospinal excitability. In our study the subjects were instructed not to observe the stretching. Nevertheless, the effect of attention to the movement throughout the experimental procedure cannot be completely excluded. In addition, passive movements repeated with the same amplitude and velocity for a certain time, might induce attempted movement, thereby activating neurons in M1 [61] and resulting in increased MEPs during DF for both TA and SOL muscles.

Despite the precise mechanisms responsible for the increased excitability cannot be clarified with this study, the findings may have clinical potential in neurorehabilitation, where passive movements are commonly applied in physiotherapy. For example in patients with paresis of the lower extremity passive dorsi- and plantar flexions can be repeatedly performed using robotic devices. It is expected that the augmented proprioceptive input facilitating motor cortical excitability, may promote motor activation and thus improve motor recovery.

## Limitations

For methodological requirements the TMS hot spot was located for stimulating the SOL, and we did not perform separate stimulations targeting specifically the TA. Nevertheless, we could observe differential MEP changes also in TA.

Additionally, the inevitable methodological requirement of repeating the stretching procedure several times, could have influenced muscle stiffness with related effects on muscle afferent feedback and proprioception.

In conclusion, it was shown that passive dynamic dorsiflexion of the plantar flexors and their static stretching facilitated the MEP after TMS recorded from both, the stretched and the shortened muscles.

This facilitation did not show a time-dependent trend or a tendency to recover during the stretching period.

## Supporting information

S1 File (XLSX)

S2 File (XLSX)
